# Microsurgical Vitrectomy with Pars Plana Incision for the Removal of Posterior Segment Intraocular Foreign Bodies

**DOI:** 10.1155/2024/3270197

**Published:** 2024-03-09

**Authors:** Xin Liu, Meng Meng Ji, Ling Jin, Ai Ping Zeng

**Affiliations:** Department of Ophthalmology, Union Hospital, Tongji Medical College, Huazhong University of Science and Technology, Wuhan 430022, China

## Abstract

This study describes a pars plana incision surgical technique combined with 23 or 25-gauge vitrectomy in the management of intraocular foreign bodies (IOFBs) and to assess its anatomical and functional results. Sixteen patients with ocular trauma complicated with IOFB were enrolled in our study. The mean preoperative visual acuity was 2.01 ± 0.55 LogMAR, and the mean postoperative visual acuity at the final visit was improved to 0.91 ± 0.58 LogMAR (*p* < 0.001). Until the last follow-up, all IOFBs were successfully removed and anatomic success was obtained. Complications, such as endophthalmitis, silicone oil-dependent, and ocular hypotonia, were not observed. Microsurgical vitrectomy with modified pars plana incision is a safe and effective procedure in the treatment of retained IOFB, especially associated with transparent lens and posterior segment injury.

## 1. Introduction

Ocular trauma often leads to severe vision loss and is an important public health problem [1, 2]. Ocular trauma with retained intraocular foreign body (IOFB) is the main cause of ocular morbidity and blindness in the working-age population [3–5]. Studies have reported that an IOFB may be present in 18% to 41% of cases of penetrating injuries of the globe [6, 7]. Ocular trauma caused by IOFB is often accompanied by penetrating injury of corneal or sclera, hyphema, traumatic cataract, vitreous haemorrhage, retinal tear or detachment, and even endophthalmitis [8]. In addition to initial damage, secondary injury-caused ocular complications during the foreign body removal process can lead to poor vision outcomes [9]. Several techniques or methods for IOFB removal have been reported by different investigators [10–16]. At present, there is no unified standard for surgical methods of foreign body removal. Among patients with different entrance and characteristics of posterior segment IOFB, choosing a suitable incision and pathway to remove the IOFB poses a unique surgical challenge to the ophthalmologist.

To explore the safety and effectiveness of modified pars plana incision in patients with retained IOFBs, here we report a series of cases treated by 23 or 25-gauge pars plana vitrectomy (PPV) in order to assess its anatomical and functional results.

## 2. Materials and Methods

### 2.1. Study Design and Subjects

In this retrospective case series study, 16 eyes from 16 patients who adopted 23 or 25-G PPV with modified pars plana incision for the removal of IOFB in Union Hospital, Tongji Medical College, Huazhong University of Science and Technology, Wuhan, China, between January 2019 and January 2022 were enrolled in the study group. The principles of this study are consistent with the Declaration of Helsinki and were approved by the Union Hospital's local ethics committee (No. 0153).

### 2.2. Examination Protocol

A thorough medical history was taken in all cases. Best-corrected visual acuity (BCVA), slit-lamp inspection of anterior segment, and posterior segment observation by indirect ophthalmoscopy were performed to assess the eye injuries. Auxiliary examinations, such as computed tomography (CT), were used to evaluate the localisation of the IOFB and assess the state of ocular trauma. All cases underwent 23 or 25-gauge PPV by the same surgeon. According to the clinical manifestation, lens extraction (lensectomy or phacoemulsification), intraocular lens (IOL) implantation, retinal repair, and other treatments were performed. Follow-up assessments were performed at 1, 3, and 6 months. Patients with incomplete data or less than 6 months of follow-up were excluded.

### 2.3. Surgical Procedures

All patients had primary wound repair or self-sealed wounds. Subsequently, a standard 23 or 25 gauge three-port vitrectomy was performed, using the vitrector with a cut rate of 5000/min (Constellation Surgical Vitrectomy System, Alcon Inc., USA). The operations were performed under retrobulbar anaesthesia, with phacoemulsification if necessary. In white cataracts, the anterior capsule was stained with 0.1% indocyanine green for a better view. Using a 6 mm long infusion cannula was considered when choroid detachment was present. All patients were treated with preservative-free triamcinolone acetonide (TA) to help complete posterior vitreous detachment (PVD) and ensure more thorough vitreous removal.

Core vitrectomy was performed before the foreign body was identified. If a foreign body is found embedded in the retina, 2-3 rows of retinal barrier laser were performed around it. With 20 gauge forceps or with the help of magnet, the IOFB was grasped along its longest axis and removed through the modified midline pars plana sclera incision (parallel to and 3.5–4 mm behind the limbus at 12 o`clock, which can preserve trocar position for continued vitrectomy) (details shown in the supplementary surgical video https://video.weibo.com/show?fid=1034:4915925892399132). For large and nonmagnetic foreign bodies, perfluorocarbon fluids may be used to protect the macula. The periphery retina was carefully evaluated at the end of surgery. A long-term intraocular tamponade may be considered if necessary. Antibiotics and steroid eye drops were administered routinely for 4 weeks with gradual tapering after surgery.

### 2.4. Main Outcome Measures

The main results were recorded and analysed including age, sex, laterality, characteristics of IOFB (size, location, and chemical nature), the time interval between injury and removal of IOFB, correlated ocular lesions, parameters related to treatment (BCVA and intraocular pressure), and complications. We considered the complete retina attachment until the last follow-up as anatomical success, the improvement in postoperative visual acuity as a parameter of functional success, and the presence of retinal detachment, ocular hypotonia, and/or endophthalmitis as complications.

### 2.5. Statistical Analyses

For statistical analysis, all numerical data are described either as mean ± standard deviation or as median (minimum-maximum). The categorical data are recorded as the number and percentage (*n*, %). The *T*-test was used for variable comparison. Visual acuity was converted into LogMAR units using an international standard. Nonnumerical vision counting fingers (CF), hand motion (HM), light perception (LP), and nonlight perception (NLP) were converted to LogMAR values of 1.9, 2.3, 2.7, and 3.0, respectively [17]. A *p* value less than 0.05 was considered as statistically significant.

## 3. Results

### 3.1. Demographical Results

Sixteen eyes from 16 patients were included in the study (shown in Table 1). All patients were men. The left eye was affected in 9/16 (56.3%) of cases. The average age was 42.1 ± 10.3 years (range, 26 to 56 years) and they were followed for 18 ± 6.9 (min: 10, max: 32, median: 18) months. All injuries to the patients were related to work accidents. None of the patients wore protective glasses at the time of injury. The interval between the injury and the removal surgery of IOFB was 6.6 ± 6.9 days (time range: 1–30 d).

### 3.2. Preoperative Accompanying Diseases

Preoperative accompanying diseases included traumatic cataract with capsular rupture in 6 (37.5%) patients, vitreous haemorrhage in 13 (81.3%) patients, retinal detachment in 9 (56.3%) patients, and endophthalmitis in 0 (0%) cases. The IOFB entrance wound site at cornea in 7 eyes (43.8%), sclera in 6 eyes (37.5%), and limbus in 3 eyes (18.7%). Except for two patients who self-sealed, the remaining 14 (87.5%) patients received primary wound closure prior to IOFB removal surgery.

### 3.3. Surgical Procedures

Table 2 summarises the general data related to the surgical procedures of the entire study population. As intraocular tamponade, nine (56.3%) patients had silicone oil tamponade and all of them completed the silicone oil extraction successfully for a mean of 5.3 months (ranging from 3 to 9 months), while the remaining seven (43.7%) patients did not need any tamping throughout the follow-up period. Among all patients, two aphakic (one had intraocular lens dislocation into the vitreous) cases (18.2%) underwent intraocular lens suspension combined with vitrectomy and IOFB resection. The remaining 14 cases were phakic eyes at the time of injury and 6 of them had traumatic cataract with lens capsular rupture. These six patients (37.5%) underwent lensectomy at the same time as PPV. They did not implant the IOL during the first surgery, in order to calculate the IOL power more accurately once the condition of the eye improved and stabilized. Four of them underwent secondary intraocular lens implantation (three suspension and one sulcus implantation). Among the eight remaining transparent lens patients, seven (43.7%) of them did not need cataract surgery until the last review. The other one underwent phacoemulsification and intraocular lens implantation when silicone oil was removed. The IOFB was magnetic in 13 patients (81.3%) and nonmagnetic in 3 patients (18.7%). The average size of the IOFBs was 2.2 mm in width (range: 1.5–3 mm) and 5.1 mm in length (range: 2–10 mm).

### 3.4. Postoperative Complications

No haemorrhage, choroidal detachment, endophthalmitis, or other significant complications were detected in the early postoperative period. At follow-up, a (6.2%) patient underwent reoperation for recurrent retinal detachment and a (6.2%) patient underwent macular epiretinal membrane peeling combined with silicone oil extraction and air tamponade. Two (12.5%) of the silicone oil tamponade patients had transient elevated intraocular pressure which can be well controlled by drug therapy alone. None of the patients had an IOP less than 6 mmHg or eyeball atrophy during follow-up.

### 3.5. Anatomical Results and Visual Outcomes

The baseline visual acuity of the patients was light perception in 2 cases (12.5%), hand movement in 6 cases (37.5%), finger count in 6 cases (37.5%), and ≥1.0 LogMAR in 2 cases (12.5%). Mean preoperative BCVA was 2.01 ± 0.55 LogMAR, and mean postoperative BCVA at the last follow-up visit was improved to 0.91 ± 0.58 LogMAR (*p* < 0.001). Among all patients, 15 cases (93.8%) showed an improvement in final vision compared to preoperative, with only one eye (6.2%) showing a decrease. Distribution of baseline and final visit BCVA is shown in Table 2. At the last follow-up, all IOFBs were successfully removed and all patients were anatomically successful.

## 4. Discussion

Intraocular foreign bodies are common in work-related trauma, and 66% of IOFB injuries occur between 21 and 40 years of age [11]. As reported in other studies, most of our patients were young men of productive age, with impairments primarily related to occupational activities [18]. None of the patients in our study wore any form of eye protection, which also highlights the need for prevention education. Traumatic eye injuries associated with IOFB may cause devastating tissue destruction and severe vision loss. The prognosis varies greatly depending on a series of factors, including preoperative status (initial visual acuity, preoperative retinal detachment, vitreous haemorrhage, and endophthalmitis), degree of injury (nature of IOFB, wound entrance, and location of IOFB), ensuing complications, and especially surgical management (duration between injury and IOFB removal and selection of surgical incision and method) [19].

The objective of IOFB management is to restore the integrity of the eyeball and maximise visual function. Due to its ability to provide direct observation and controlled surgical procedures, PPV is currently the primary surgical method for removing posterior segment IOFBs. In recent years, with the development of minimally invasive vitreoretinal surgical instruments and techniques, microsurgical vitrectomy provides higher cutting efficiency and more stable intraocular pressure control, which can reduce the incidence of secondary complications [20, 21]. Currently, there is no consensus on the incision for IOFB removal due to the diversity of trauma conditions. In most cases, the IOFB was removed by incision in the corneosclera or through an enlarged trocar opening, which may not maintain the advantages of microsurgical PPV during subsequent surgical procedures [11].

In the present study, we assessed the safety and efficacy of 23 or 25-gauge vitrectomy with modified pars plana incision for the management of posterior segment IOFB. The mean BCVA was significantly improved from 2.01 ± 0.55 LogMAR to 0.91 ± 0.58 LogMAR (*p* < 0.001) and anatomical success was achieved in 100% of the cases. Compared to light perception to 20/80 before surgery, visual acuity improved to CF/1 m to 20/33 at the final follow-up. BCVA was improved in all patients, except for one patient whose foreign body was removed 1 month later due to delayed discovery (patient no. 3).

The timing of IOFB removal is one of the key factors affecting the clinical prognosis. In some cases, corneal edema and turbidity may interfere with the visibility of the posterior segment, and PPV must be postponed until corneal edema resolves. Poor observation clarity may affect surgical outcomes and increase the risk of iatrogenic lesions, incomplete IOFB resection, and inadequate management of associated ocular injuries, but delayed surgery reduces the chance of visual recovery and increases the risk of complications such as proliferative vitreoretinopathy (PVR), toxic reaction, and endophthalmitis [22, 23]. Although no endophthalmitis occurred in our study, we recommend that immediate vitrectomy surgical resection should be considered if endophthalmitis is suspected; otherwise, it can be delayed until corneal edema improves and intraocular inflammation is better controlled. According to our experience, these processes usually take around 7 days. In addition, if the conditions are met, we do not recommend that the foreign body be removed too late. For our only case where vision did not improve, it may be directly related to the secondary epimacular membrane caused by the excessive delay in IOFB removal (patient no. 3). No cases of endophthalmitis were observed in our study. On the one hand, it may be attributed to the prophylactic use of vancomycin (1 mg/0.1 ml) and ceftazidime (2 mg/0.1 ml) intra-vitreous during primary suture. On the other hand, it also reflects the safety and effectiveness of microsurgical vitrectomy with pars plana incision for IOFB removal.

Traumatic cataract is a common complication of IOFB patients, with a reported incidence of 44%∼66% [9]. In our study, the incidence of traumatic cataract was 37.5%. In order for the posterior segment to be clearly visible, cataract removal is necessary in this case. However, in a significant proportion of cases (our study was 50%), a minor damage to the lens may lead to local nonprogressive lens opacity and may not require surgery [16]. In our study, all eight patients retained clear lenses when foreign bodies were removed. And seven of them did not need cataract surgery until their last review. The results show that the modified incision has certain advantages for patients with transparent crystalline lens.

At present, retinal detachment is the principal cause of visual loss after ocular trauma [24]. Despite current advances in treatment technology, retinal detachment before or after surgery remains a common and devastating complication. PVR is the main impact factor on retinal detachment after trauma [19, 25, 26]. IOFB-related retinal detachment occurs in 16 to 47% of patients [27, 28]. In this study, preoperative retinal detachment occurred in 9 (56.3%) patients. Except for one case of recurrence of retinal detachment caused by PVR due to excessive IOFB (patient no. 4), the rest of the patients were successfully reattached. None of the other 7 patients with normal retina before surgery was observed to have retinal detachment during follow-up. This also demonstrated the safety of the modified pars plana incision combined with microsurgical PPV for the removal of IOFB. Removal of foreign bodies through a modified pars plana incision did not have an adverse effect on the scleral trocar incision. After closing the pars plana incision, the advantages of minimally invasive PPV surgery can be retained and stable intraocular pressure can be maintained, providing a guarantee for the treatment of subsequent lesions.

Where indications are available, we do not recommend removing IOFB without vitrectomy due to the risk of vitreous incarceration and the possible traction damage to intraocular tissues. Shah et al. found that in the process of removing foreign bodies, perfluorocarbon fluids can be used to protect the macula to prevent foreign body shedding and damage [29]. In our experience, there was no shedding during the removal of IOFB in all 16 patients, and only in one case of nonmagnetic foreign body, we used perfluorocarbon solution protection.

**Figure 1:**
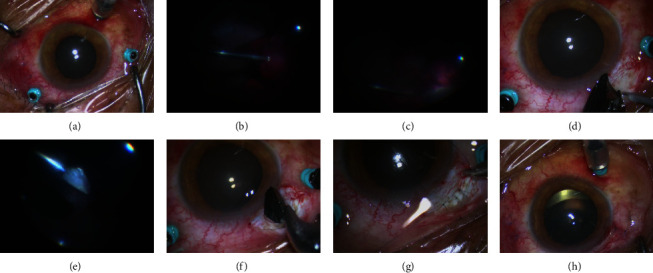
Patient no. 10 with transparent lens preoperative (a), combined with vitreous hemorrhage (b), clearance of accumulated blood to exposure of foreign body (c), modified pars plana incision was made (d), removal IOFB through modified incision with the help of magnet (e and f), close the modified incision to maintain intraocular stability for continued vitrectomy (g), lens keep transparent at the end of the operation (h).

**Figure 2:**
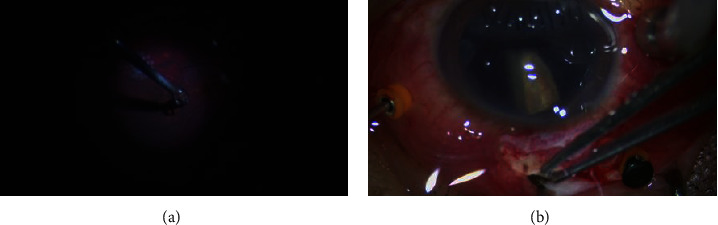
Modified incision suitable for IOFBs of different sizes and shapes, patient no. 8 with small size IOFB (a) and patient no. 4 with large size IOFB (b).

In this present study, to remove IOFB, we performed a modified pars plana incision parallel to and 3.5–4 mm behind the limbus at 12 o`clock to preserve trocar position for continued vitrectomy (details shown in the supplementary surgical video https://video.weibo.com/show?fid=1034:4915925892399132). We did not need to enlarge the trocar incision, which avoided incision leakage during the treatment of subsequent lesions and maintained stable intraocular pressure, thus keeping the advantage of minimally invasive surgery. This modified pars plana incision has advantage for patients with intact lens (shown in Figure 1), and it is suitable for IOFBs of different sizes and shapes (shown in Figure 2). Compared with clear cornea or limbus tunnel incision, modified incision through pars plana has less adverse effect on corneal endothelium and lens capsule and will not increase the complications like iatrogenic retinal hole or haemorrhage. The main limitations of this study include its noncomparative and retrospective design. In order to compare our technique with other methods previously established, randomized controlled studies with larger sample sizes are required.

## 5. Conclusions

Nonetheless, in conclusion, microsurgical vitrectomy with a modified pars plana incision is a safe and effective method for the treatment of posterior segment IOFBs, especially associated with transparent crystalline lens and posterior segment injury. Our procedure showed considerable functional success and low rates of complication.

## Figures and Tables

**Table 1:** Characteristics of the patients.

Demographic and clinical features (*n* = 16)		
Age	Years, mean ± SD (range)	42.1 ± 10.3 (26–56)

Gender	Female	0
	Male	16

Eye	Right	43.7%
	Left	56.3%

Interval	Days, mean ± SD (range)	6.6 ± 6.9 (1–30)

Follow-up	Months, mean ± SD (range)	18 ± 6.9 (10–32)

**Table 2:** General data for the entire study population.

Patient no.	Age (years)	IOFB entrance site	Size of IOFB (mm)	Property of IOFB	Time to IOFB removal (days)	Time to cataract surgery (days)	Lens status	VH	Retina tear or detachment (tamponade)	Baseline BCVA (LogMAR)	Final BCVA (LogMAR)	Follow-up(months)
1	26	C/I/L	2 *∗* 3 *∗* 5	Magnetic	4	4	Totally opaque	+	−	2.3	0.2	25
2	47	C/I/SL	3 *∗* 5 *∗* 6	Magnetic	1	Not done	Cortex cataract	+	+ silicone oil	2.3	1.9	30
3	55	Limbal/SL	2 *∗* 2 *∗* 3	Magnetic	30	90	Cortex cataract	−	+ silicone oil	0.6	1.4	24
4	48	C/I/L	3 *∗* 5 *∗* 10	Magnetic	2	2	Totally opaque	+	+ silicone oil	2.3	1.3	21
5	54	C/I/L	2 *∗* 2 *∗* 3	Nonmagnetic	11	11	Totally opaque	+	+	2.7	1.9	20
6	32	Limbal	2 *∗* 8 *∗* 10	Nonmagnetic	7	Aphakic	Aphakic	−	−	1.9	0.5	20
7	50	Sclera	2 *∗* 4 *∗* 7	Magnetic	7	Not done	Cortex cataract	+	+ silicone oil	1.9	0.9	15
8	56	Sclera	1.5 *∗* 2 *∗* 2	Nonmagnetic	7	Aphakic	Aphakic	+	−	1	0.7	15
9	30	C/L	3 *∗* 5 *∗* 6	Magnetic	1	1	Totally opaque	+	+ silicone oil	2.7	1.9	32
10	49	Sclera	2 *∗* 3 *∗* 5	Magnetic	7	Not done	Clear	+	+ silicone oil	1.9	0.4	14
11	32	C/L	2 *∗* 2 *∗* 3	Magnetic	1	1	Totally opaque	+	+ silicone oil	1.9	0.6	14
12	38	Sclera	2 *∗* 2 *∗* 3	Magnetic	7	Not done	Cortex cataract	+	+	1.9	0.6	14
13	42	Limbal	2 *∗* 2 *∗* 3	Magnetic	3	Not done	Cortex cataract	−	−	1.9	0.4	12
14	50	C/L	3 *∗* 4 *∗* 5	Magnetic	4	4	Totally opaque	+	−	2.3	0.7	12
15	28	Sclera	2 *∗* 2 *∗* 5	Magnetic	7	Not done	Clear	+	+ silicone oil	2.3	0.5	10
16	36	Sclera	2 *∗* 3 *∗* 5	Magnetic	7	Not done	Clear	+	+ silicone oil	2.3	0.6	10

C: cornea; I: iris; L: lens; SL: suspensory ligament; IOFB: intraocular foreign body; VH: vitreous haemorrhage; BCVA: best-corrected visual acuity.

## Data Availability

The datasets used and/or analysed during the current study are available from the corresponding author on reasonable request.
